# Supplementary material for: Aurora-A mediated phosphorylation of LDHB promotes glycolysis and tumor progression by relieving the substrate-inhibition effect
Source: Nat Commun. 2019 Dec 5;10:5566. doi: 10.1038/s41467-019-13485-8 (PMC6895051; doi:10.1038/s41467-019-13485-8)
Supplement: Supplementary file 1 — Supplementary Information [file 41467_2019_13485_MOESM1_ESM.pdf]

Supplementary information for the manuscript entitled:

Aurora-A Mediated Phosphorylation of LDHB promotes glycolysis and tumor progression by relieving the substrate-inhibition effect

Cheng et al.

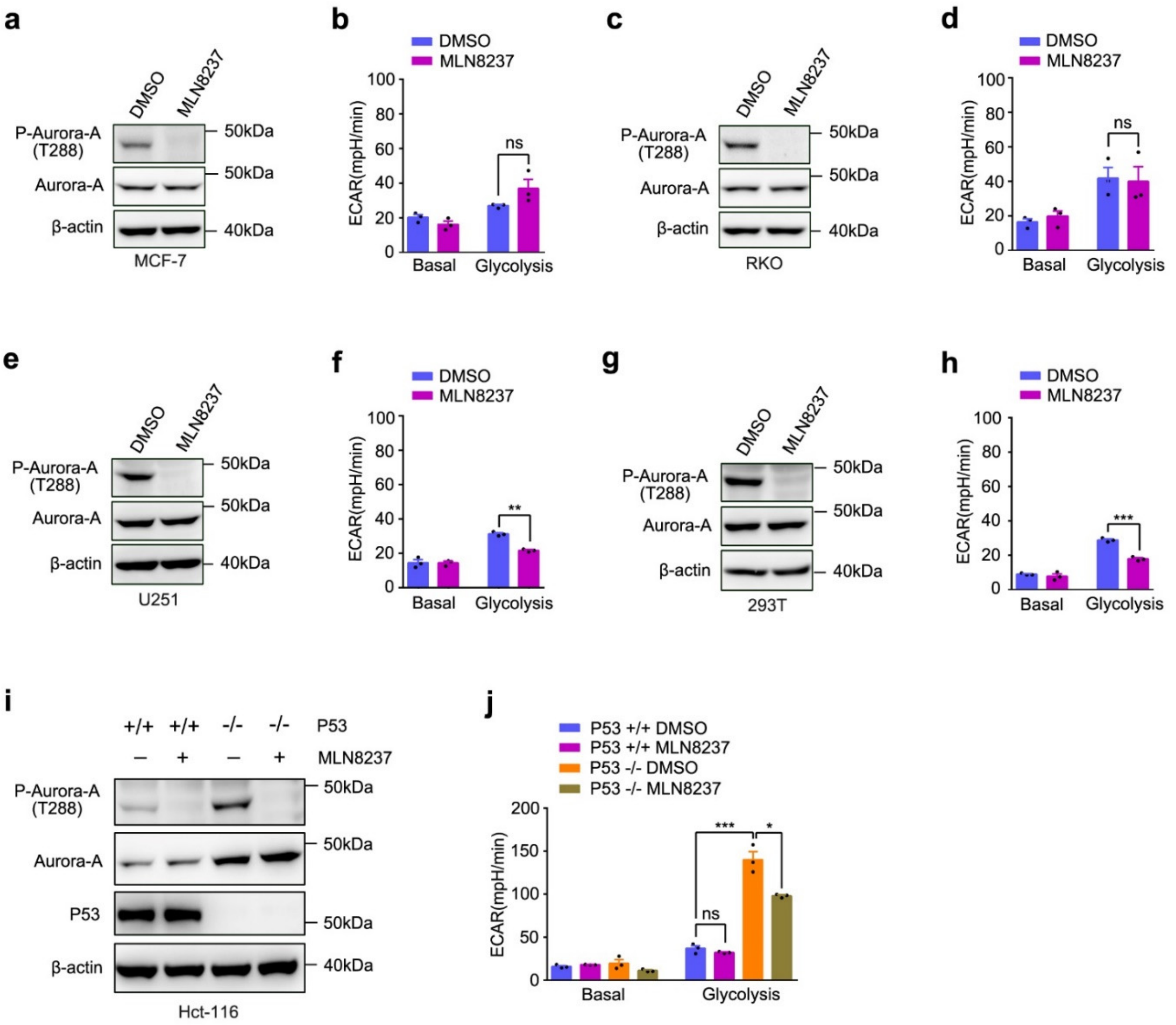

Supplemental Figure 1: Aurora-A promotes glycolysis in p53-deficient cancer cells. (a) MCF-7 cells were treated with DMSO or MLN8237. The levels of Aurora-A and p-Aurora-A (T288ph) were examined by WB. **(b)** The glycolytic rates of cells in **(a)** were investigated using seahorse assay. **(c)** RKO cells were treated with DMSO or MLN8237. The levels of Aurora-A and p-Aurora-A were examined by WB. **(d)** The glycolytic rates of cells in **(c)** were investigated using seahorse assay. **(e)** U251 cells were treated with DMSO or MLN8237. The levels of Aurora-A and p-Aurora-A were examined by WB. **(f)** The glycolytic rates of cells in **(e)** were investigated using seahorse assay. **(g)** 293T cells were treated with DMSO or MLN8237. The levels of Aurora-A and p-Aurora-A were examined by WB. **(h)** The glycolytic rates of cells in **(g)** were investigated using seahorse assay. **(i)** HCT116 p53 -/- and HCT116 p53+/+ cells were treated with DMSO or MLN8237. The levels of Aurora-A, p-Aurora-A and p53 were examined by WB. **(j)** The glycolytic rates of cells in **(i)** were investigated using seahorse assay. The error bar in panels **(b)**, **(d)**, **(f)**, **(h)**, **(j)** represents the SEM, n=3 biological independent samples. Source data are provided as a Source Data file.(Student t-test * $p<0.05$, ** $p<0.01$, *** $p<0.001$, ns: not significant)

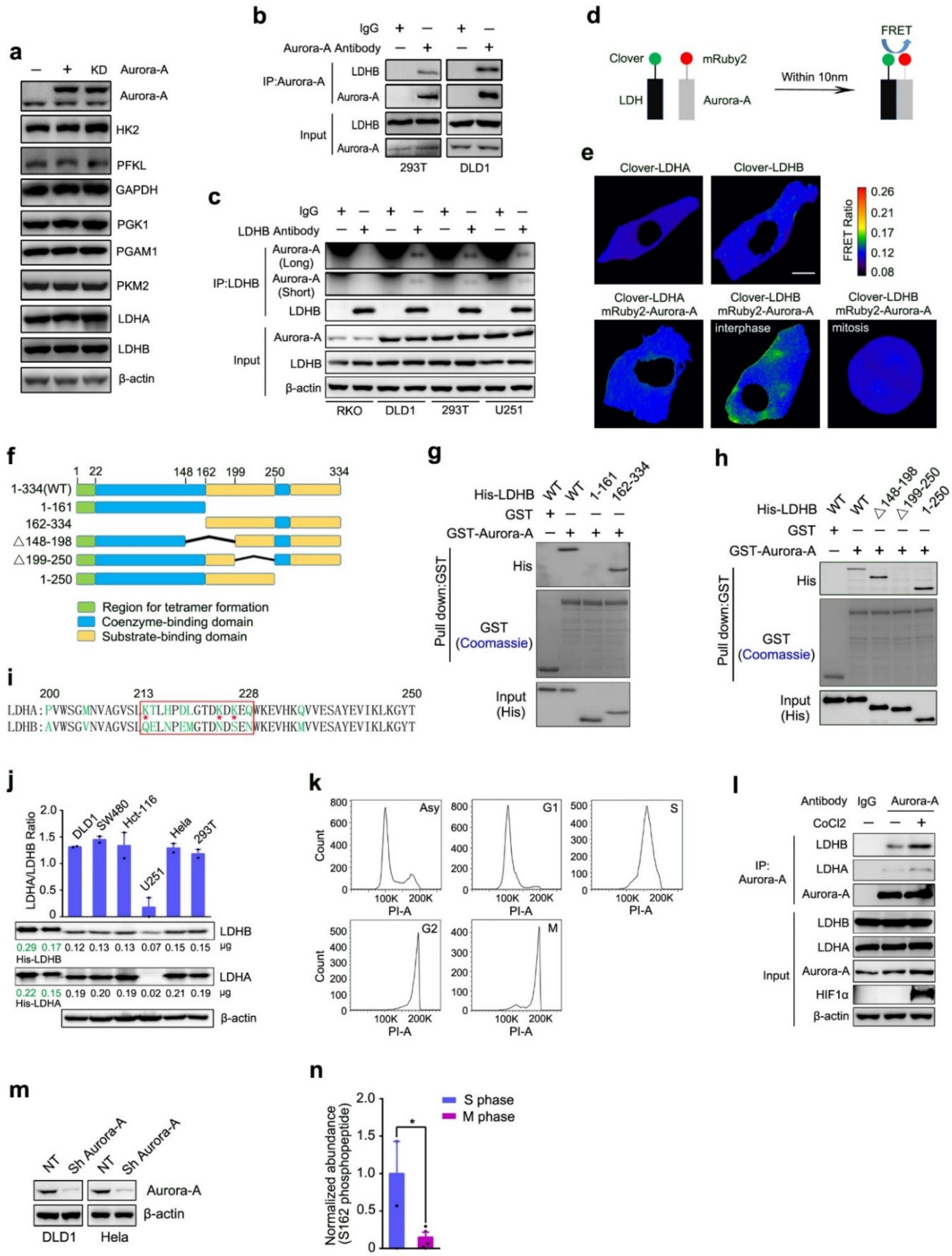

Supplemental Figure 2: Aurora-A directly binds to LDHB. **(a)** Empty vector (-), wild type Aurora-A (+) and KD Aurora-A (KD) were expressed in HCT116 cells. The expressions of glycolytic enzymes were examined by WB. **(b)** Endogenous Aurora-A interacts with LDHB in 293T and DLD1 cells. **(c)** Endogenous LDHB interacts with Aurora-A in 293T, DLD1 and U251 cells. **(d)** Schematic diagram of FRET assay to determine the direct interaction between Clover-LDHA/B and mRuby2-Aurora-A in single cells. **(e)** Clover-LDHA/B and mRuby2-Aurora-A were

transfected into DLD1 cells. The ratio images of FRET assay in cells expressing LDHA/B and/or Aurora-A were shown. Scale bar, 10 μm. **(f)** The schematic diagrams of the functional domains, and deletions or truncation mutants of LDHB. **(g)** GST pull-down assay was performed with GST-Aurora-A and His-tagged LDHB N-terminus (aa 1-161) or C-terminus (aa 162-334) to narrow down the interaction domain that mediates the interaction between LDHB and Aurora-A. **(h)** GST pull-down assay was performed with GST-Aurora-A and His-tagged LDHB deletion mutants illustrated in **(f)**. **(i)** Amino acids in the region of LDHB that is essential for the interaction with Aurora-A. **(j)** Purified his-tagged LDHA/B with known quantity were subjected to WB with lysates from different cells. The ratio was calculated. **(k)** The cell cycle stages after synchronization were analyzed by FACS for cells used in Fig. **2e**. Asy: asynchronized population. **(l)** DLD1 was treated with Cocl2 (200 μM, 12 hr) to induce the expression of HIF1a. Co-IP was conducted with Aurora-A antibody. **(m)** WB showed Aurora-A was depleted in DLD1 and Hela cells in Fig. **2k**. **(n)** Endogenous LDHB was isolated for MS analysis to identify phosphorylation residues. The relative abundance of S162 phosphopeptide was quantified. The error bar in panels **(j)**, **(n)** represents the SEM, n=2 biological independent samples in panels **(j)**, n=2 biological independent samples for S phase, n=4 for M phase in panels **(n)**. Source data are provided as a Source Data file.(Student t-test * $p<0.05$)

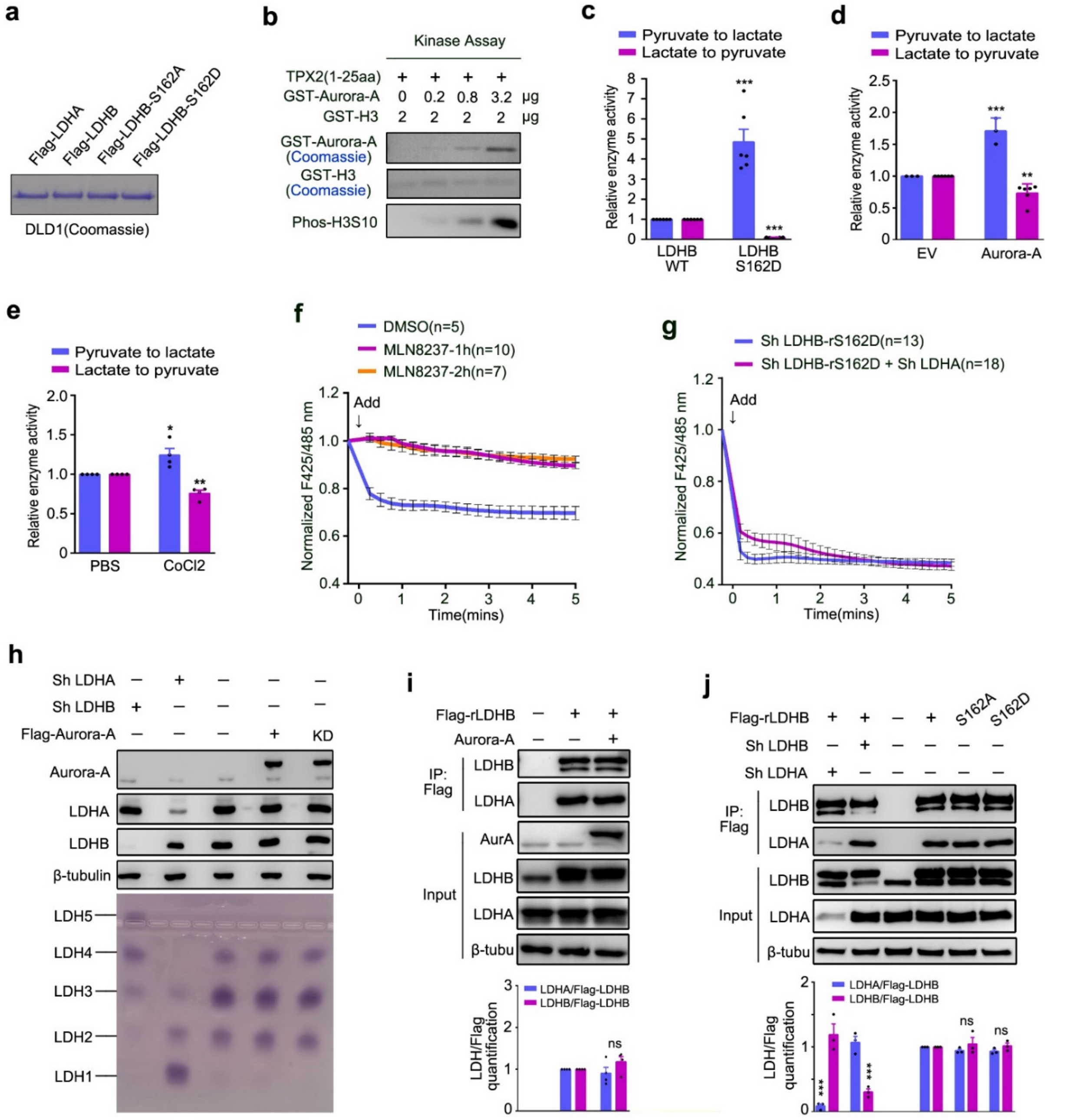

Supplemental Figure 3: Phosphorylation of LDHB S162 alters its enzymatic activities. (a) The LDH proteins in DLD1 cells used in Fig. **3c** were purified with FLAG beads. Eluted proteins were subjected to SDS-PAGE and Coomassie blue staining. **(b)** The recombinant GST-Aurora-A was allosteric activated using peptide of TPX2 1-25 amino acids. The activity of Aurora-A was evaluated by the phosphorylation of Histone H3S10. **(c)** In U251 cells, endogenous LDHB was knocked-down, then shRNA-resistant and FLAG-tagged wild type LDHB and LDHB S162D were expressed. Proteins were purified and subjected to measurements of bidirectional activities. **(d)** Aurora-A or empty vector (EV) were transfected into the U251 cells used in **(c)**. FLAG-LDHB was isolated by IP and subjected to measurements of bidirectional activities. **(e)** DLD1 cells treated with CoCl2 for 12 hours. FLAG-LDHB was isolated by IP and subjected to measurements of bidirectional activities. **(f)** NADH/NAD$^+$ sensor SoNar was transfected into DLD1 cells. Aurora-A was inhibited with MLN8237 for 1 or 2 hours, the ratios of F425/485 were measured in live cells. **(g)** SoNar was transfected into DLD1 cells used in Fig. **3g**. LDHA was knocked down. The ratios of F425/485 were measured in live cells.

(h) In DLD1 cells, LDHA/B was depleted by shRNA or Aurora-A WT/KD was overexpressed. The expressions of proteins were detected by WB. LDH isozymes (LDH1-LDH5) were visualized in gel after native gel electrophoresis. **(i)** FLAG-tagged LDHB was transfected or co-transfected with Aurora-A into DLD1 cells. Co-IP was performed with FLAG-beads followed by WB to examine the affinity between endogenous LDHA/B and exogenous LDHB. **(j)** In DLD1 cells, LDHA or LDHB was knocked-down by shRNA and LDHB WT /S162A/D mutants were transfected as indicated. Co-IP was conducted with FLAG beads and followed by WB to examine the affinity between endogenous LDHA/B and exogenous LDHB WT/Mutants. The error bar in panels **(c)**, **(d)**, **(e)**, **(i)**, **(j)** represents the SEM, n=6 biological independent samples in panels **(c)**, n=3 biological independent samples in panels **(d)**, **(e)**, **(i)**, **(j)**. Source data are provided as a Source Data file.(Student t-test * $p<0.05$, ** $p<0.01$, *** $p<0.001$, ns: not significant.)

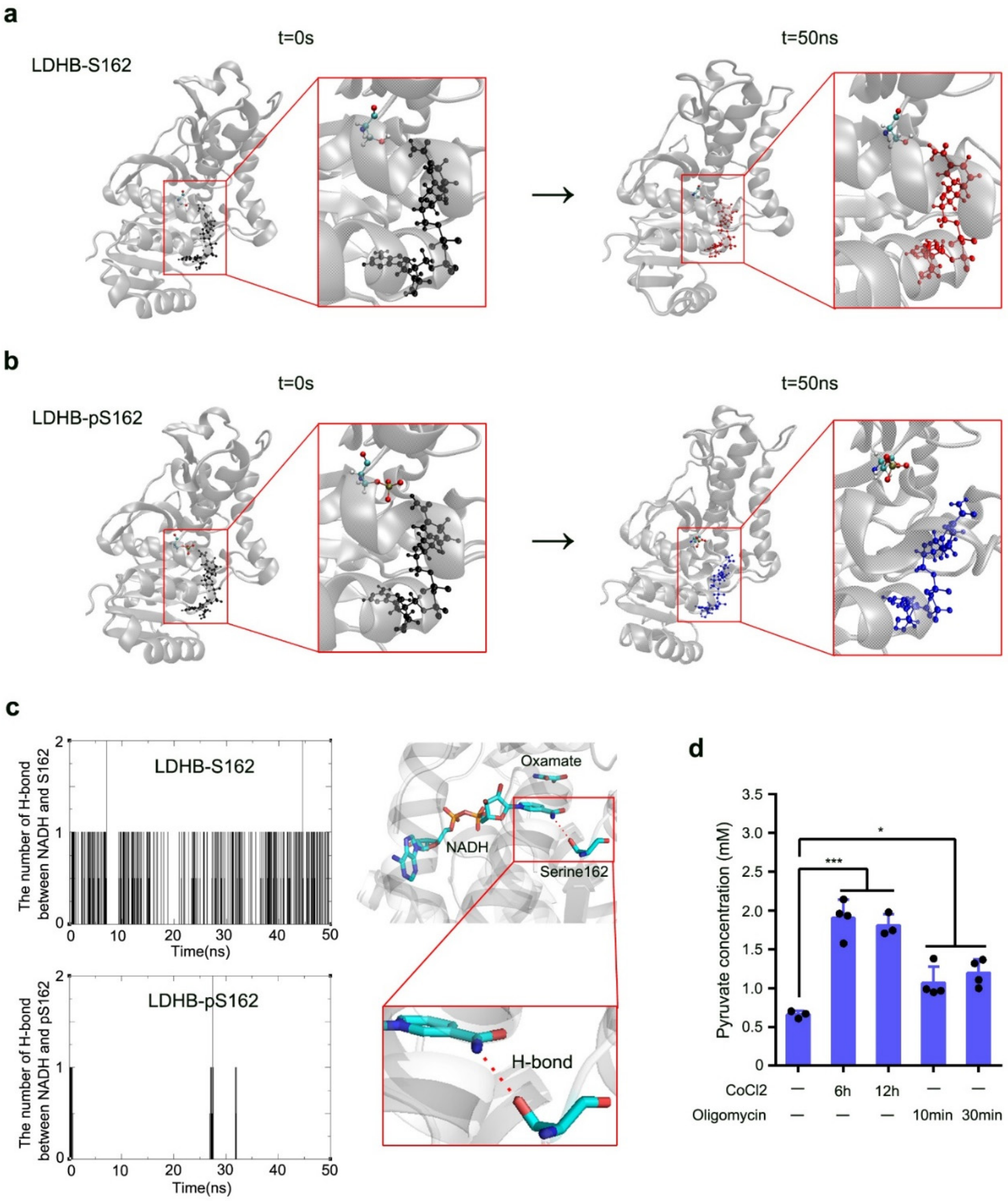

Supplemental Figure 4: Phosphorylation of LDHB S162 relieves substrate inhibition. (a) Molecular modeling revealed the dynamic conformational changes for wild type LDHB and co-enzyme NADH in 50 ns. The first and the last conformations are shown. S162 of LDHB and NADH are colored. Enlarged views of the active center are shown in red frames. **(b)** Molecular modeling for LDHB and NADH after LDHB serine162 is phosphorylated. The first and the last conformations in 50 ns simulations are shown. Enlarged views of phosphorylated S162 and NADH are shown in red frames. **(c)** The numbers of hydrogen bonds (right panel) between nicotinamide ring of NADH and LDHB S162 wild type (upper panel) or LDHB pS162 (lower panel) were extracted from the molecular dynamics stimulation, and were plotted over 50 nanosecond simulation. **(d)** The concentrations of intracellular pyruvate in DLD1 cells were measured after treatment with DMSO, CoCl2 (200 μM) and Oligomycin (1 μM). The error bar in panels **(d)** represents the SEM, n=3 biological independent samples. Source data are provided as a Source Data file.(Student t-test * p<0.05, ** p<0.01, *** p<0.001)

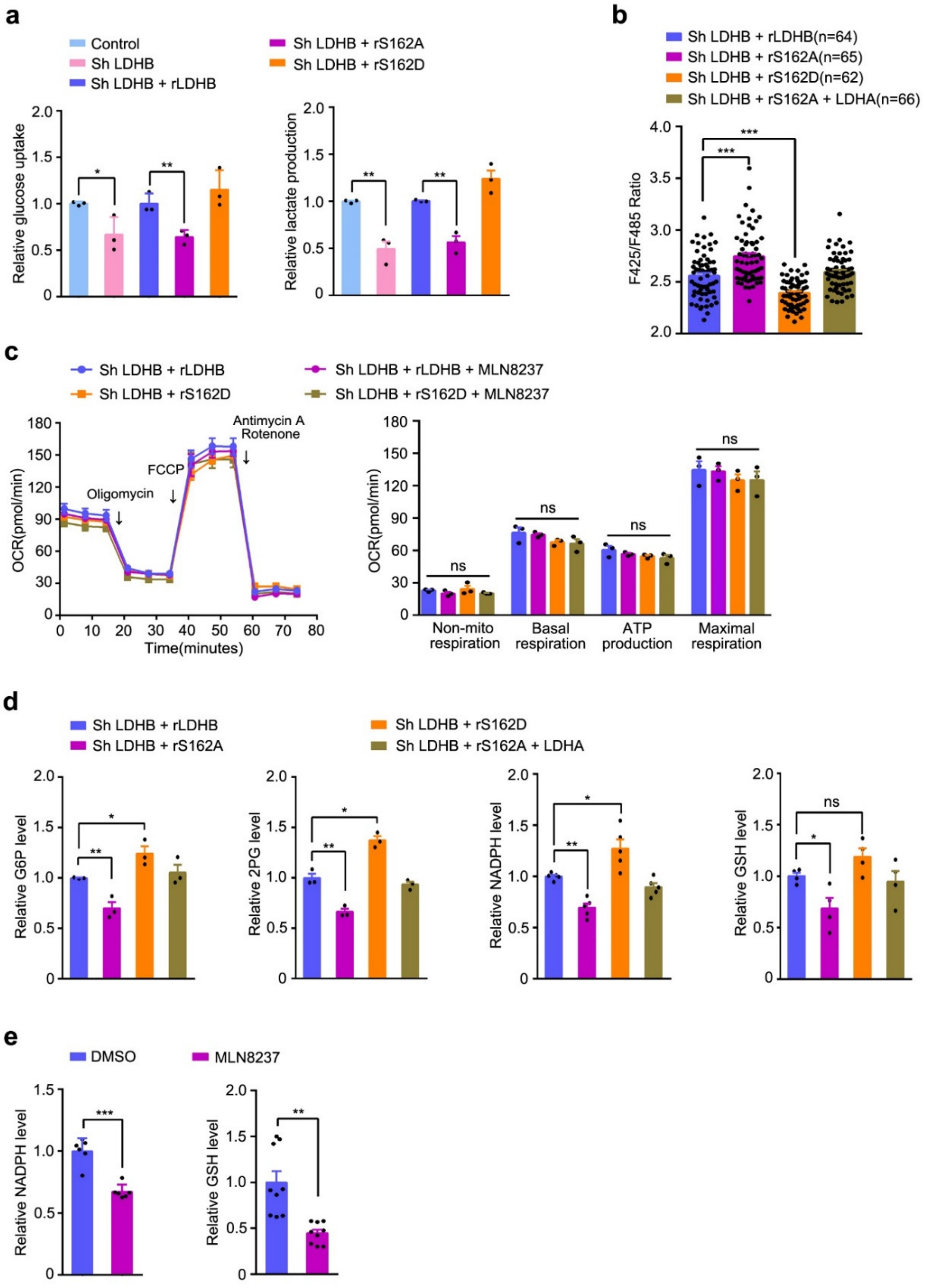

Supplemental Figure 5: LDHB phosphorylation promotes glycolysis and biosynthesis. (a) LDHB was knocked down in DLD1 cells, and sh RNA resistant LDHB wild type, or S162A/D mutants were expressed. The relative glucose uptake (left panel) and lactate production (right panel) were measured. **(b)** DLD1 cells used in Fig. **5c** were transfected with SoNar sensor. The ratios of F425/F485 were measured in L15 medium. **(c)** The oxygen consumption rates (OCR) were analyzed using seahorse assay in the same DLD1 cells as used in Fig. **5e**. The OCR over time (left panel) and OCR in each stage of measurements were shown (right panel). Non-mitochondria respiration, basal respiration, ATP production and maximal respiration were calculated.

(d) The levels of several glycolytic intermediate metabolites glucose-6-phosphate (G6P), 2-phosphoglycerate (2PG) and products of biosynthesis pathways NADPH, GSH were determined in DLD1 cells used in Fig. **5c**. **(e)** The levels of NADPH (left panel) and GSH (right panel) were measured in DLD1 cells after treatment with DMSO or MLN8237 (200 nM, 4 hr). The error bar in panels **(a)**, **(c)**, **(d)**, **(e)** represents the SEM, n=3 biological independent samples in panels **(a)**, **(c)**, **(d)**, n=6 biological independent samples in panels **(e)**. Source data are provided as a Source Data file.(Student t-test * $p<0.05$, ** $p<0.01$, *** $p<0.001$, ns: not significant)

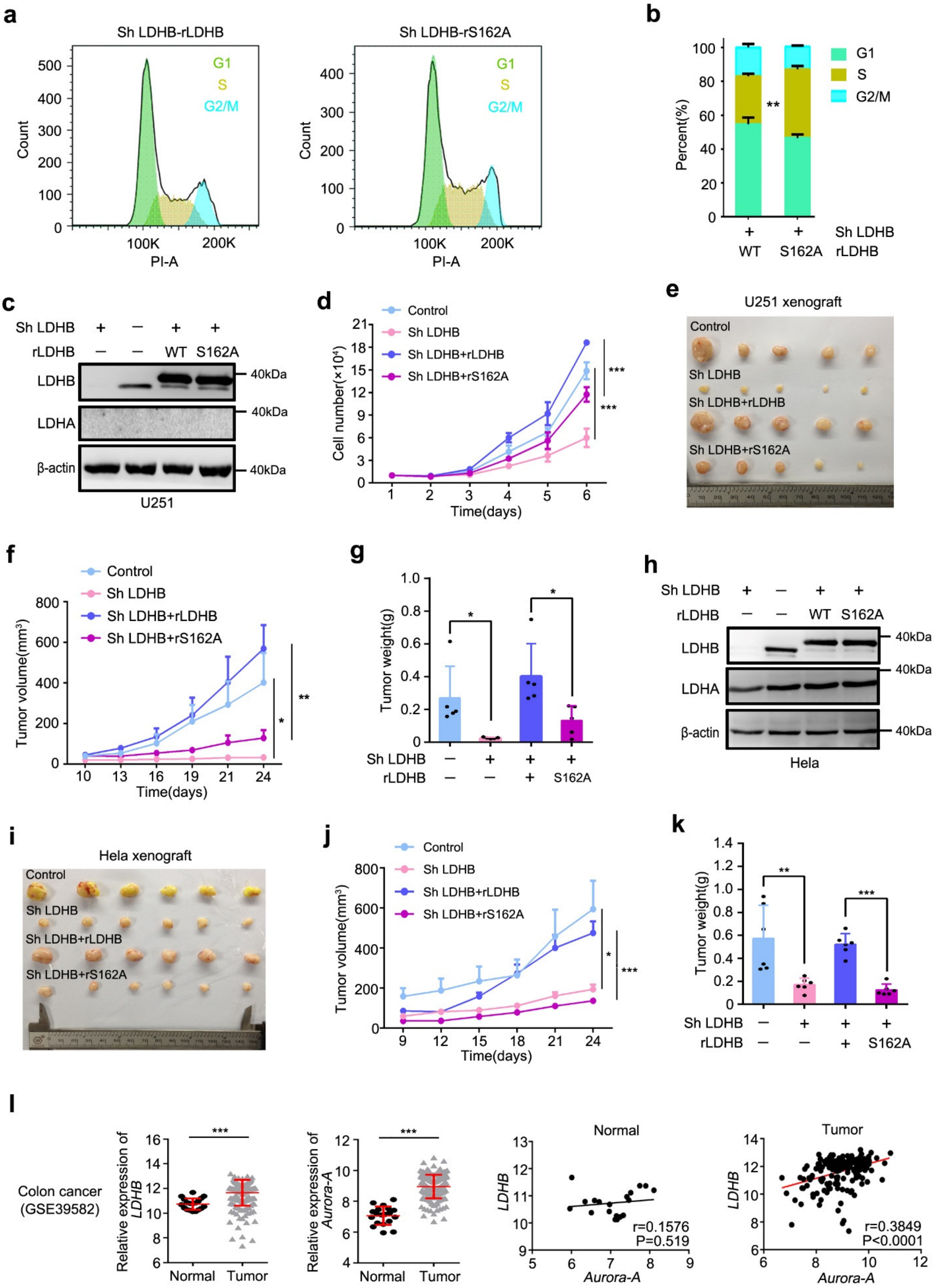
a
Sh LDHB-rLDHB
Sh LDHB-rS162A
G1
S
G2/M
Count
PI-A
100K
200K
b
Percent(%)
G1
S
G2/M
**
Sh LDHB
rLDHB
WT
S162A
c
Sh LDHB
rLDHB
WT
S162A
LDHB
LDHA
β-actin
40kDa
U251
d
Control
Sh LDHB
Sh LDHB+rLDHB
Sh LDHB+rS162A
Cell number(×10⁴)
Time(days)
***
e
U251 xenograft
Control
Sh LDHB
Sh LDHB+rLDHB
Sh LDHB+rS162A
f
Control
Sh LDHB
Sh LDHB+rLDHB
Sh LDHB+rS162A
Tumor volume(mm³)
Time(days)
*
**
g
Tumor weight(g)
Sh LDHB
rLDHB
S162A
*
h
Sh LDHB
rLDHB
WT
S162A
LDHB
LDHA
β-actin
40kDa
Hela
i
Hela xenograft
Control
Sh LDHB
Sh LDHB+rLDHB
Sh LDHB+rS162A
j
Control
Sh LDHB
Sh LDHB+rLDHB
Sh LDHB+rS162A
Tumor volume(mm³)
Time(days)
*
***
k
Tumor weight(g)
Sh LDHB
rLDHB
S162A
**
***
l
Colon cancer
(GSE39582)
Relative expression of LDHB
Relative expression of Aurora-A
Normal
Tumor
***
Normal
Tumor
LDHB
Aurora-A
r=0.1576
P=0.519
r=0.3849
P<0.0001

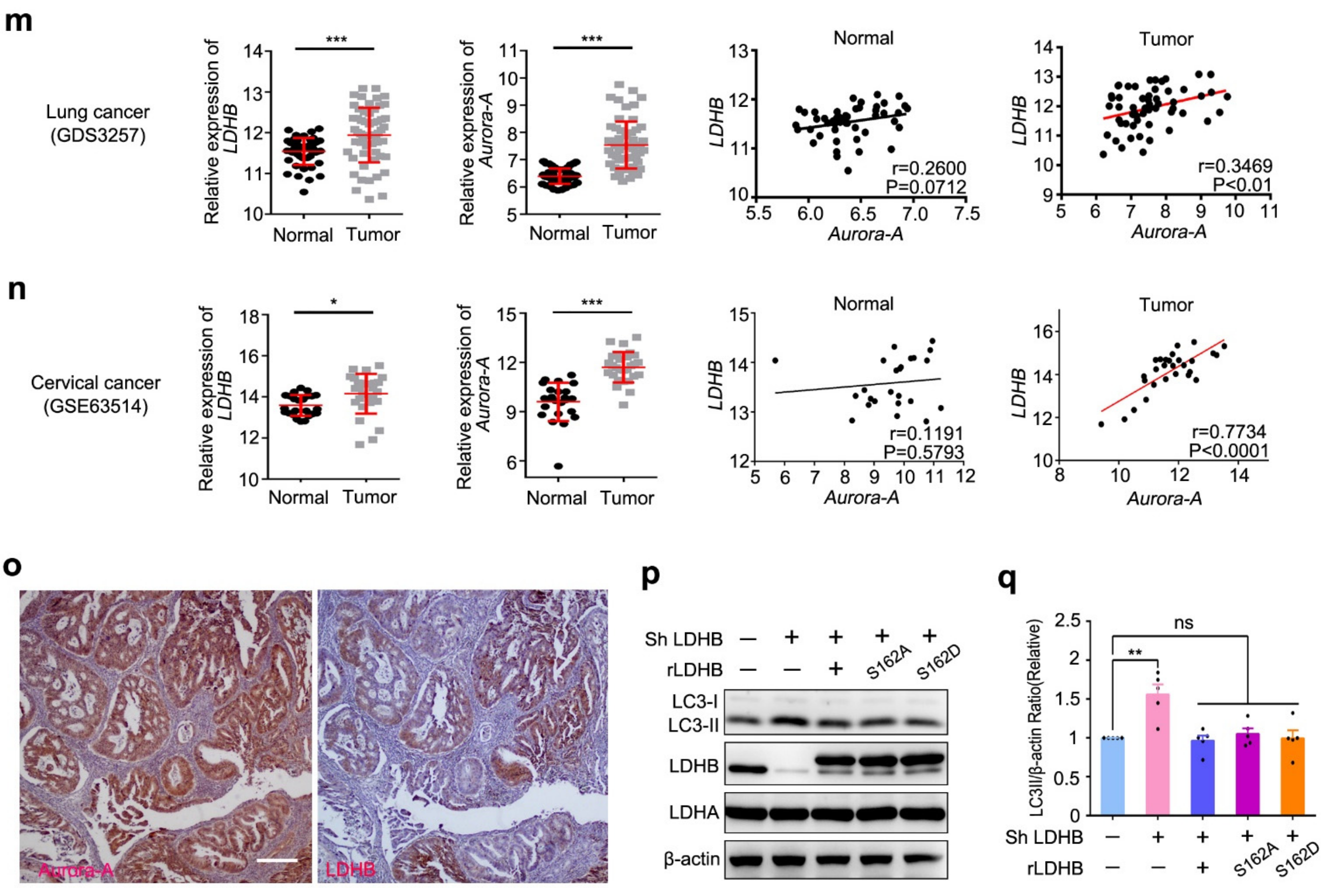

Supplemental Figure 6: Phosphorylation of LDHB is required for tumor growth. (a) DLD1 cells used in Fig.**6a** were subjected to FACS analysis. Cell cycle profiles were showed. **(b)** The ratios of G1, S and G2/M phase of cells in **(a)** were shown. **(c)** In U251 cells endogenous LDHB was knocked down, then shRNA-resistant wild type LDHB and LDHB S162A were expressed. **(d)** The growth curve of U251 cells used in **(c)**. **(e)** U251 cells used in **(c)** were inoculated in nude mice. Xenograft tumors at the end point were collected and shown. **(f)** The growth curve of the tumors in **(e)**. **(g)** The weight of tumors in **(e)**. **(h)** In Hela cells, endogenous LDHB was knocked down, then shRNA-resistant wild type LDHB and LDHB S162A were expressed. **(i)** Hela cells used in **(h)** were inoculated in nude mice. The tumors at the endpoint were shown. **(j)** The growth curve of the tumors in **(i)**. **(k)** The weight of tumors in **(i)**. **(l)** The correlation between the expression of Aurora-A and LDHB in clinical colon samples with p53 mutation were analyzed. Data from GSE39582. **(m)** The correlation between Aurora-A and LDHB expression in normal lung and lung cancer tissues were analyzed. Data from GDS33257. **(n)** The correlation between Aurora-A and LDHB expression in normal cervical and cervical cancer tissues were analyzed. Data from GSE63514. **(o)** Immuno-histochemical staining of consecutive tumor sections showed that Aurora-A and LDHB are simultaneously over-expressed in human colon cancer samples. Scale bar, 250 μm. **(p)** DLD1 cells used in Fig.**6a**. were starved in HBSS for 0.5 hour. The expressions of LC3-II were examined by WB. **(q)** The levels of LC3-II were quantified to evaluate the status of autophagy in **(p)**. The error bar in panels **(b)**, **(d)**, **(q)** represents the SEM, The error bar in panels **(f)**, **(g)**, **(j)**, **(k)** represents the SD. n=3 biological independent samples in panels **(b)**, **(d)**. n=5 biological independent samples in panels **(f)**, **(g)**, **(q)**. n=6 biological independent samples in panels **(j)**, **(k)**. Source data are provided as a Source Data file.(Student t-test * $p<0.05$, ** $p<0.01$, *** $p<0.001$, ns: not significant)

Commercial antibodies used for this study

Antigene	Company	Reference	Dilution
Flag	TransGen Biotech	HT201	1:10000
HA	Proteintech	66006-1-Ig	1:5000
His	TransGen Biotech	HT501	1:1000
GST	Proteintech	66001-1-Ig	1:5000
LDHA	Proteintech	19987-1-AP	1:5000
LDHB	Proteintech	19988-1-AP	1:5000
LDHB	Abcam	ab75167	1:5000
GAPDH	Proteintech	60004-1-Ig	1:5000
β-Tubulin	Sigma	T4026	1:5000
β-actin	Proteintech	66009-1-Ig	1:5000
LC3	Proteintech	12135-1-AP	1:2500
Aurora A	BD Transduction	610939	1:1000
Aurora A	Cell Signaling Technology	4718	1:1000
Aurora-A-pT288	Cell Signaling Technology	3079	1:1000
Hif1α	Proteintech	20960-1-AP	1:1000
Pan-phosphoserine	Abcam	ab9332	1:100

Primers used in this study

Primer name	Primer sequence
His/HA-LDHA-F	GGAAGATCTATGGCAACTCTAAAGGA
His/HA-LDHA-R	CGGAATTCTTAAAATTGCAGCTCCT
His/HA-LDHB-F	CGGGATCCATGGCAACTCTTAAGGA
His/HA-LDHB-R	CGGAATTCTCACAGGTCTTTTAGGT
Flag-LDHB-F	GAAGATCTGATGGCAACTCTTAAGGA
Flag-LDHB-R	GGGGTACCTCACAGGTCTTTTAGGT
Flag-LDHA-F	AGATCTGATGGCAACTCTAAAGGA
Flag-LDHA-R	GGTACCTTAAAATTGCAGCTCCT
Clover-LDHB-F	GCtctagaATGGCAACTCTTAAGGAAA
Clover-LDHB-R	GCtctagaTCACAGGTCTTTTAGGTCC
Clover-LDHA-F	GCtctagaATGGCAACTCTAAAGGATC
Clover-LDHA-R	GCtctagaTTAAAATTGCAGCTCCTTT
mRuby2-Aurora A-F	CGGGATCCGATGGACCGATCTAAAGAAA
mRuby2-Aurora A-R	CGGGATCCacAGACTGTTTGCTAGCTG
GST-Aurora-A-F	CGGGATCCTGATGGACCGATCTAAAG
GST-Aurora-A-R	CCCTCGAGCTAAGACTGTTTGCT
His-LDHB 1-161-R	CGGAATTCTCATCCAATCACGCGGTGTT
His-LDHB 162-334-F	CGGGATCCAGTGGATGTAATCTGGA
His-LDHB 1-250-R	CGGAATTCTCAGTTGGTATATCCTTTTA
His-LDHB Del 148-198-F	CTTACGTATGTTGTGGCTGTGTGGAGTGG
His-LDHB Del 148-198-R	CCACACAGCCACAACATACGTAAGAATGT
His-LDHB Del 199-250-F	GGCGACTCAAGTTGGGCTATTGGATTAAG
His-LDHB Del 199-250-R	TCCAATAGCCCAACTTGAGTCGCCATGTT
LDHB S162A overlap-F	GCGTGATTGGAGCTGGATGTAATCTG
LDHB S162A overlap-R	CAGATTACATCCAGCTCCAATCACGC
LDHB S162D overlap-F	GCGTGATTGGAGACGGATGTAATCTG
LDHB S162D overlap-R	CAGATTACATCCGTCTCCAATCACGC
