# Supplementary material for: Aurora-A mediated phosphorylation of LDHB promotes glycolysis and tumor progression by relieving the substrate-inhibition effect
Source: Nat Commun. 2019 Dec 5;10:5566. doi: 10.1038/s41467-019-13485-8 (PMC6895051; doi:10.1038/s41467-019-13485-8)
Supplement: Supplementary file 3 — Description of Additional Supplementary Files [file 41467_2019_13485_MOESM3_ESM.pdf]

Description of Additional Supplementary Files

File Name: Supplemental Table 1

Description: MS results of proteins that were identified from FLAG-Aurora-A IP-MS experiment (Fig. 2a).

File Name: Supplemental Table 2

Description: Quantification of phosphorylated peptides identified from MS analysis (Fig. 2k and Supplemental Fig. 2n). Twenty worksheets are included.
