# Supplementary material for: Aurora-A mediated phosphorylation of LDHB promotes glycolysis and tumor progression by relieving the substrate-inhibition effect
Source: Nat Commun. 2019 Dec 5;10:5566. doi: 10.1038/s41467-019-13485-8 (PMC6895051; doi:10.1038/s41467-019-13485-8)
Supplement: Supplementary file 6 — Reporting Summary [file 41467_2019_13485_MOESM6_ESM.pdf]

nature research

Corresponding author(s): Zhenye Yang
Last updated by author(s): Nov 2, 2019

Reporting Summary

Nature Research wishes to improve the reproducibility of the work that we publish. This form provides structure for consistency and transparency in reporting. For further information on Nature Research policies, see Authors & Referees and the Editorial Policy Checklist.

Statistics

For all statistical analyses, confirm that the following items are present in the figure legend, table legend, main text, or Methods section.

n/a	Confirmed	
☐	☒	The exact sample size (n) for each experimental group/condition, given as a discrete number and unit of measurement
☐	☒	A statement on whether measurements were taken from distinct samples or whether the same sample was measured repeatedly
☐	☒	The statistical test(s) used AND whether they are one- or two-sided<br>*Only common tests should be described solely by name; describe more complex techniques in the Methods section.*
☒	☐	A description of all covariates tested
☒	☐	A description of any assumptions or corrections, such as tests of normality and adjustment for multiple comparisons
☐	☒	A full description of the statistical parameters including central tendency (e.g. means) or other basic estimates (e.g. regression coefficient) AND variation (e.g. standard deviation) or associated estimates of uncertainty (e.g. confidence intervals)
☐	☒	For null hypothesis testing, the test statistic (e.g. F, t, r) with confidence intervals, effect sizes, degrees of freedom and P value noted<br>*Give P values as exact values whenever suitable.*
☒	☐	For Bayesian analysis, information on the choice of priors and Markov chain Monte Carlo settings
☒	☐	For hierarchical and complex designs, identification of the appropriate level for tests and full reporting of outcomes
☒	☐	Estimates of effect sizes (e.g. Cohen's d, Pearson's r), indicating how they were calculated

Our web collection on statistics for biologists contains articles on many of the points above.

Software and code

Policy information about availability of computer code

Data collection	Statistical data: GraphPad Prism (version6.0)<br>Living cell imaging:softWoRx(version6.5.2)<br>Seahorse:Wave(Version2.3.0)
Data analysis	Statistical analysis: GraphPad Prism (version 6.0)<br>Molecular dynamics (MD) analysis: VMD software (version 1.94a12) and Amber software (version 16).<br>Isothermal Titration Calorimetry (ITC) analysis: MicroCal Origin software (version 7.0).<br>Immunofluorescence staining and FRET analysis: Image J software (version 1.8.0)

For manuscripts utilizing custom algorithms or software that are central to the research but not yet described in published literature, software must be made available to editors/reviewers. We strongly encourage code deposition in a community repository (e.g. GitHub). See the Nature Research guidelines for submitting code & software for further information.

Data

Policy information about availability of data

All manuscripts must include a data availability statement. This statement should provide the following information, where applicable:

- Accession codes, unique identifiers, or web links for publicly available datasets
- A list of figures that have associated raw data
- A description of any restrictions on data availability

The mass spectrometry proteomics data have been deposited to the ProteomeXchange Consortium via the PRIDE partner repository with the dataset identifier PXD016110(Figure 2a).
All other data supporting the findings of this study are available from the corresponding author on reasonable request.

Field-specific reporting

Please select the one below that is the best fit for your research. If you are not sure, read the appropriate sections before making your selection.

☒ Life sciences ☐ Behavioural & social sciences ☐ Ecological, evolutionary & environmental sciences

For a reference copy of the document with all sections, see nature.com/documents/nr-reporting-summary-flat.pdf

Life sciences study design

All studies must disclose on these points even when the disclosure is negative.

Sample size	The chosen sample size are based on the numbers used for previous publications, which is most optimal to generate statistically significant results. No statistical methods were used to predetermine sample sizes.
Data exclusions	No samples or animals were excluded from the analyses.
Replication	All replicates are biological replicates obtained from biologically independent experiments. All attempts at replication were successful. The experiments number has been clearly stated in the figure legends.
Randomization	The samples/cells were randomized to be examined. The mice were randomized to put into separate groups /cages for experiments.
Blinding	For all experiments, the investigators were divided into two groups. One group were blinded to allocation during experiments and outcome assessment.

Reporting for specific materials, systems and methods

We require information from authors about some types of materials, experimental systems and methods used in many studies. Here, indicate whether each material, system or method listed is relevant to your study. If you are not sure if a list item applies to your research, read the appropriate section before selecting a response.

Materials & experimental systems

n/a	Involved in the study	
☐	☒	Antibodies
☐	☒	Eukaryotic cell lines
☒	☐	Palaeontology
☐	☒	Animals and other organisms
☒	☐	Human research participants
☒	☐	Clinical data

Methods

n/a	Involved in the study	
☒	☐	ChIP-seq
☐	☒	Flow cytometry
☒	☐	MRI-based neuroimaging

Antibodies

Antibodies used	mouse anti-Flag (TransGen Biotech, Beijing, China; HT201,1:5000);rabbit or mouse anti-HA (Proteintech, Wuhan, China; 66006-1-Ig,1:5000);mouse anti-His (TransGen Biotech, Beijing, China; HT501,1:1000);mouse anti-GST (Proteintech, Wuhan, China; 66001-1-Ig,1:5000); rabbit anti-LDHA (Proteintech, Wuhan, China; 19987-1-AP,1:5000);rabbit anti-LDHB (Proteintech, Wuhan, China; 19988-1-AP,1:5000 and Abcam, Cambridge, MA, USA; ab75167,1:5000);mouse anti-GAPDH (Proteintech, Wuhan, China; 60004-1-Ig,1:10000), mouse anti-β-Tubulin(Sigma, St Louis, MO,USA; T4026,1:5000),mouse anti-β-actin (Proteintech, Wuhan, China; 66009-1-Ig,1:5000);rabbit anti-LC3 (Proteintech, Wuhan, China; 12135-1-AP,1:2500), rabbit or mouse anti-Aurora A (BD Transduction, Waltham, MA, USA; 610939,1:1000 and Cell Signaling Technology, Danvers, MA, USA; 4718,1:2500);Rabbit anti-Aurora-A-pT288 (Cell Signaling Technology, Danvers, MA, USA; 3079,1:1000);rabbit anti-phosphoserine (Abcam, Cambridge, MA, USA; ab9332,1:200);rabbit anti-Hif1(Proteintech, Wuhan, China; 20960-1-AP,1:1000)
Validation	All commercial antibodies were validated by manufacturers for indicated species and applications.

Eukaryotic cell lines

Policy information about cell lines

Cell line source(s)	A549, MCF-7, RKO, 293T, DLD1, Hela, U251 and Hct-116 were obtained from cell library of the Chinese Academy of Sciences.
Authentication	Cells were authenticated using the short tandem repeat (STR) method.
Mycoplasma contamination	All cell lines were routinely tested negative for mycoplasma contamination.

Commonly misidentified lines
(See ICLAC register)

No commonly misidentified cell lines were used.

Animals and other organisms

Policy information about studies involving animals; ARRIVE guidelines recommended for reporting animal research

Laboratory animals	BALB/C athymic nude mice, male, 6 weeks.
Wild animals	The study did not involve wild animals.
Field-collected samples	The study did not involve samples collected from the field.
Ethics oversight	All animals were kept in specific pathogen-free conditions. All experimental procedures involving mice were carried out as prescribed by the National Guidelines for Animal Usage in Research (China) and were approved by the Ethics Committee at the University of Science and Technology of China.

Note that full information on the approval of the study protocol must also be provided in the manuscript.

Flow Cytometry

Plots

Confirm that:

- [ ] The axis labels state the marker and fluorochrome used (e.g. CD4-FITC).
- [ ] The axis scales are clearly visible. Include numbers along axes only for bottom left plot of group (a 'group' is an analysis of identical markers).
- [ ] All plots are contour plots with outliers or pseudocolor plots.
- [ ] A numerical value for number of cells or percentage (with statistics) is provided.

Methodology

Sample preparation	*Describe the sample preparation, detailing the biological source of the cells and any tissue processing steps used.*
Instrument	*Identify the instrument used for data collection, specifying make and model number.*
Software	*Describe the software used to collect and analyze the flow cytometry data. For custom code that has been deposited into a community repository, provide accession details.*
Cell population abundance	*Describe the abundance of the relevant cell populations within post-sort fractions, providing details on the purity of the samples and how it was determined.*
Gating strategy	*Describe the gating strategy used for all relevant experiments, specifying the preliminary FSC/SSC gates of the starting cell population, indicating where boundaries between "positive" and "negative" staining cell populations are defined.*

- [ ] Tick this box to confirm that a figure exemplifying the gating strategy is provided in the Supplementary Information.
